# Adipocyte-specific CD1d-deficiency mitigates diet-induced obesity and insulin resistance in mice

**DOI:** 10.1038/srep28473

**Published:** 2016-06-22

**Authors:** Masashi Satoh, Miyuki Hoshino, Koki Fujita, Misao Iizuka, Satoshi Fujii, Christopher S. Clingan, Luc Van Kaer, Kazuya Iwabuchi

**Affiliations:** 1 Department of Immunology, Kitasato University School of Medicine, Sagamihara, Japan; 2 Department of Laboratory Medicine, Asahikawa Medical University, Asahikawa, Japan; 3 Department of Emergency Medicine, Tufts Medical Center, Lowell General Hospital, Boston, MA 02111, USA; 4 Department of Pathology, Microbiology and Immunology, Vanderbilt University School of Medicine, Nashville, TN, USA

## Abstract

It has been shown that CD1d expression and glycolipid-reactive, CD1d-restricted NKT cells exacerbate the development of obesity and insulin resistance in mice. However, the relevant CD1d-expressing cells that influence the effects of NKT cells on the progression of obesity remain incompletely defined. In this study, we have demonstrated that 3T3-L1 adipocytes can present endogenous ligands to NKT cells, leading to IFN-γ production, which in turn, stimulated 3T3-L1 adipocytes to enhance expression of CD1d and CCL2, and decrease expression of adiponectin. Furthermore, adipocyte-specific CD1d deletion decreased the size of the visceral adipose tissue mass and enhanced insulin sensitivity in mice fed a high-fat diet (HFD). Accordingly, NKT cells were less activated, IFN-γ production was significantly reduced, and levels of adiponectin were increased in these animals as compared with control mice on HFD. Importantly, macrophage recruitment into the adipose tissue of adipocyte-specific CD1d-deficient mice was significantly blunted. These findings indicate that interactions between NKT cells and CD1d-expressing adipocytes producing endogenous NKT cell ligands play a critical role in the induction of inflammation and functional modulation of adipose tissue that leads to obesity.

The immune system plays an important role in controlling adipose tissue function and homeostasis^1^,^2^,^3^. Adipose tissue contains both resident and recruited immune cells, including macrophages, eosinophils, T cells and B cells^4^,^5^,^6^,^7^,^8^. Accordingly, obesity is closely associated with chronic inflammation of adipose tissue initiated by activation of these immune cells. For example, T helper (Th) 1-biased cytokine responses characterized by CD8^+^ T cells and M1 macrophages leads to insulin resistance and obesity, whereas Th2-biased cytokine responses involving eosinophils, M2 macrophages and regulatory T cells suppress adipose tissue inflammation and obesity^4^,^5^,^8^. These findings have led to the concept that the immune balance in adipose tissue is critically important in controlling adipose tissue inflammation. Therefore, understanding how different immune cells in adipose tissue are activated and communicate with each other may reveal novel methodologies to curtail obesity, inflammation and its associated health risks.

Natural killer T (NKT) cells are a unique subset of T-lineage cells that recognize glycolipid antigens in the context of CD1d molecules. NKT cells promptly produce large amounts of Th1, Th2, and Th17 cytokines upon TCR stimulation^9^,^10^. A prototype ligand, α-galactosylceramide (α-GalCer), is recognized by type I or invariant NKT (iNKT) cells that express an invariant TCR α-chain, Vα14-Jα18 in mice and Vα24-Jα18 in humans^11^. A second type of NKT cells, type II or variant NKT (vNKT) cells that express more diverse TCR appear to recognize a variety of lipid antigens (Ag) including sulfatide^12^. NKT cells are present in spleen, liver, bone marrow and thymus^10^, as well as adipose tissue^13^. Several research groups have investigated the role of the CD1d-NKT cell axis in the development of diet-induced obesity (DIO) in mice. Results obtained have been divergent, as some research groups reported no effect (neutral)^14^, protection^15^,^16^,^17^, or promotion^18^,^19^ of obesity-associated disease. The relevant cells in adipose tissue expressing CD1d that induce these effects have not been investigated. Cells in adipose tissue expressing CD1d include macrophages, dendritic cells and adipocytes.

Here, we show that 3T3-L1 adipocytes can present both exogenous and endogenous lipid antigens to NKT cells in a CD1d-dependent manner. Furthermore, we found that CD1d^f/f^-adipoq-cre mice^20^,^21^, which selectively lack CD1d expression in adipocytes, gain less body weight and exhibit improved insulin sensitivity than littermate control mice when fed a high-fat diet. Mechanisms underlying the development of obesity and insulin resistance are discussed in relation to NKT cell-adipocyte interactions.

## Results

### Adipocytes express CD1d and the co-stimulatory molecule CD80

**Figure 1:**
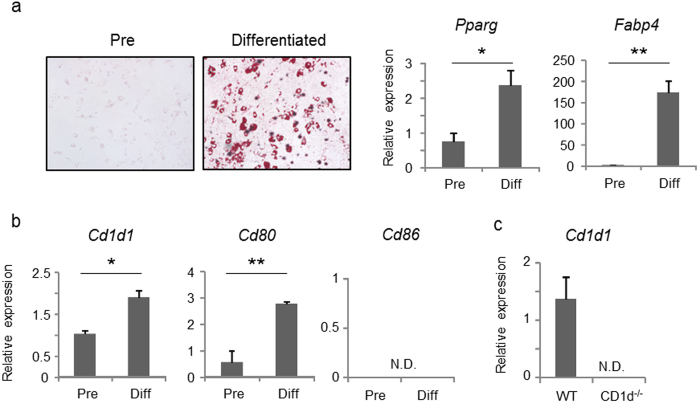
3T3-L1 adipocytes express CD1d and co-stimulatory molecules. (**a,b**) 3T3-L1 cells before (Pre) or after differentiation (Diff; 3T3-L1-adipocytes) were stained with Oil-red-O. mRNA expression of the mature adipocyte markers *Pparg* and *Fabp4* (**a**), *Cd1d1*, *Cd80* and *Cd86* (**b**), were analyzed by qPCR. (**c**) The *Cd1d1* expression of the adipocyte fraction in visceral adipose tissue from WT or CD1d^−/−^ mice was analyzed by qPCR. Representative data from at least 3 independent experiments are shown. Data are shown as mean ± s.d. Statistical analysis was performed according to the Student’s *t*-test. *P < 0.05, **P < 0.01.

To examine whether mature adipocytes can activate NKT cells, we employed 3T3-L1 fibroblasts (pre-adipocytes) differentiated to mature adipocytes *in vitro* as a model. 3T3-L1 adipocytes showed accumulation of lipid droplets, increased levels of *Pparg*, a critical regulator of adipogenesis, and increased levels of fatty acid binding protein 4 (*Fabp4*) upon differentiation (Fig. 1a). Differentiation of 3T3-L1 cells to adipocytes was accompanied by increases in *Cd1d1* expression (Fig. 1b). Expression of the co-stimulatory molecule *Cd80* was induced after differentiation, whereas *Cd86* was undetectable before and after differentiation (Fig. 1b). Furthermore, mature adipocytes collected from visceral adipose tissue of WT mice expressed significant levels of *Cd1d1* when compared with adipocytes from CD1d-deficient mice (Fig. 1c). These data indicated that adipocytes express CD1d and co-stimulatory molecules, and thus may be able to influence NKT cells.

### 3T3-L1 adipocytes present α-GalCer to iNKT cells

**Figure 2:**
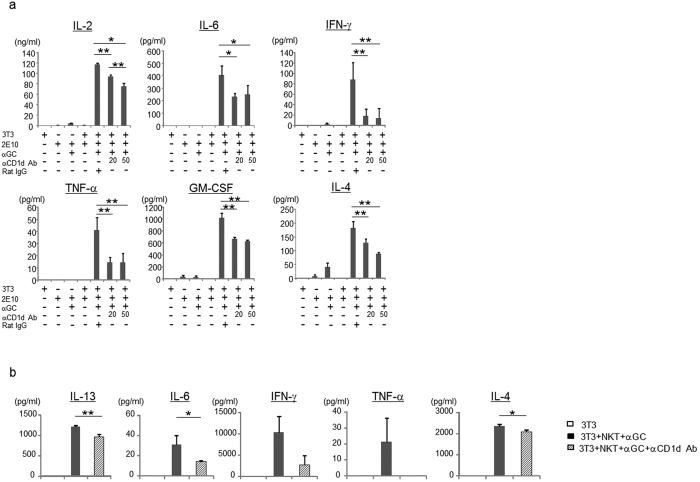
3T3-L1 adipocytes can present α-GalCer to iNKT cells through CD1d. (**a**) 2E10 hybridoma cells were co-cultured with 3T3-L1 adipocytes w/or w/o α-GalCer and anti-CD1d (α-CD1d) mAb. (**b**) Sorted NKT cells (TCRβ^+^NK1.1^+^) were co-cultured with 3T3-L1 adipocytes in the presence of α-GalCer w/or w/o α-CD1d mAb. The cytokines in the supernatant were quantified with FlowCytomix Multiplex kit. Representative data from at least 2 independent experiments are shown. Data are shown as mean ± s.d. Statistical analysis was performed according to the Tukey-Kramer test *P < 0.05, **P < 0.01.

To examine the ability of 3T3-L1 adipocytes to activate NKT cells, we cultured 2E10 iNKT hybridoma cells with 3T3-L1 adipocytes in the presence of α-GalCer. 2E10 cells responded to α-GalCer presented by 3T3-L1 adipocytes and produced IL-2, IL-6, IFN-γ, TNF-α, GM-CSF, and IL-4 (Fig. 2a), which was significantly inhibited by anti-CD1d mAb (Fig. 2a). Next, we examined whether primary NKT cells sorted as TCRβ^+^NK1.1^+^ cells containing both iNKT and vNKT cells (total NKT cells) also responded to α-GalCer-cultured 3T3-L1 adipocytes. As observed with the hybridoma cells, sorted murine NKT cells produced cytokines in a CD1d-dependent manner (Fig. 2b), indicating that 3T3-L1 adipocytes can present α-GalCer to activate murine iNKT cells via CD1d.

### Both iNKT and vNKT cells are activated by 3T3-L1 adipocytes

**Figure 3:**
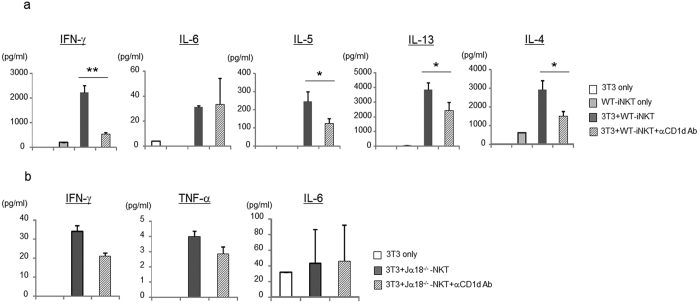
3T3-L1 adipocytes present endogenous ligands to NKT cells. (**a,b**) Sorted iNKT cells (TCRβ^+^αGC tet^+^) from WT mice (**a**) or NKT cells (TCRβ^+^NK1.1^+^) from Jα18^−/−^ mice (**b**) were co-cultured with 3T3-L1 adipocytes w/ or w/o α-CD1d Ab. The cytokines in the supernatant were quantified by FlowCytomix Multiplex kit. Representative data from at least 3 independent experiments are shown. Data are shown as mean ± s.d. Statistical analysis was performed according to the Tukey-Kramer test *P < 0.05, **P < 0.01.

Next, to examine whether adipocytes expressed endogenous lipid ligands for NKT cells *in vivo*, sorted NKT cells were cultured with 3T3-L1 adipocytes in the absence of exogenous ligand. iNKT cells which were isolated by sorting with α-GalCer/CD1d tetramers produced both Th1 and Th2 cytokines when cultured with 3T3-L1 adipocytes (Fig. 3a). It has been reported that Jα18^−/−^ mice that lack iNKT cells developed diet-induced obesity to the same extent as WT mice, whereas CD1d^−/−^ mice lacking both iNKT and vNKT cells exhibited improved metabolic parameters as compared with WT mice, suggesting that vNKT cells may also respond to endogenous Ag presented by CD1d on adipocytes^18^. Thus, we also examined the activation of sorted TCRβ^+^NK1.1^+^ cells from Jα18^−/−^ mice (this includes vNKT cells and a small subset of CD1d-independent NK1.1^+^ T cells) by 3T3-L1 adipocytes. TCRβ^+^NK1.1^+^ cells from Jα18^−/−^ mice also produced IFN-γ, TNF-α and IL-6 when cultured with 3T3-L1 cells (Fig. 3b), which was largely CD1d-dependent (Fig. 3b). Taken together, our findings indicated that 3T3-L1 adipocytes express endogenous ligand(s) that can activate both iNKT and vNKT cells.

### 3T3-L1 cells fail to express endogenous α-glycosylceramide NKT cell ligands

**Figure 4:**
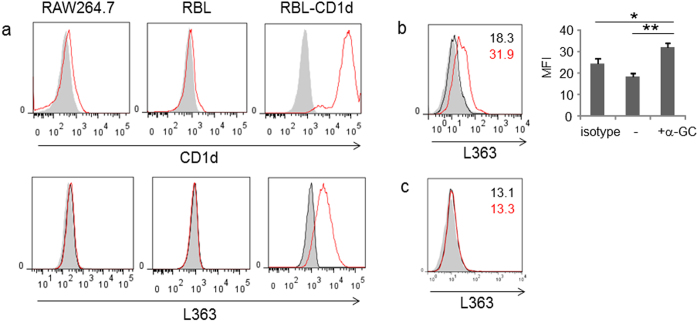
The endogenous NKT cell ligands in 3T3-L1 adipocytes. (**a**) The expression of CD1d (upper panel, gray: isotype control, red line: α-CD1d) and α-GalCer:CD1d complex (L363) (lower panel, gray: isotype, black line: no treatment, red line: α-GalCer loaded) in RAW264.7, RBL and RBL-CD1d. (**b**) The expression of α-GalCer:CD1d complex (L363) (gray: isotype, black line: no treatment, red line: α-GalCer loaded) in 3T3-L1 adipocytes. (**c**) The expression of α-GalCer:CD1d complex (L363) (gray: isotype, black line: no treatment, red line: TNF-α treatment (2 ng/ml, 20 h)) in 3T3-L1 adipocytes. Numbers shown in the panel are a value of mean fluorescence intensity (MFI) of the respective treatment (black: no treatment; red: experimental). Representative data from at least 2 independent experiments are shown. Data are shown as mean ± s.d. Statistical analysis was performed according to the Tukey-Kramer test *P < 0.05, **P < 0.01.

The above results suggested that 3T3-L1 cells could present endogenous ligands to NKT cells. Because endogenous α-GlyCer has been shown to activate iNKT cells, we employed the L363 antibody that detects α-GlyCer/CD1d complexes. While RAW264.7, RBL, RBL-CD1d, and 3T3-L1 cells failed to stain with L363 antibody, CD1d-expressing cells stained with the antibody when incubated with α-GalCer (Fig. 4a,b). Furthermore, treatment of 3T3-L1 cells with TNF-α, which has been suggested to induce α-GlyCer production in some cells^22^, was unable to enhance staining (Fig. 4c). Taken together, 3T3-L1 adipocytes likely express endogenous NKT cell ligands that are distinct from α-GlyCer.

### NKT cells produce IFN-γ in adipose tissue during obesity

**Figure 5:**
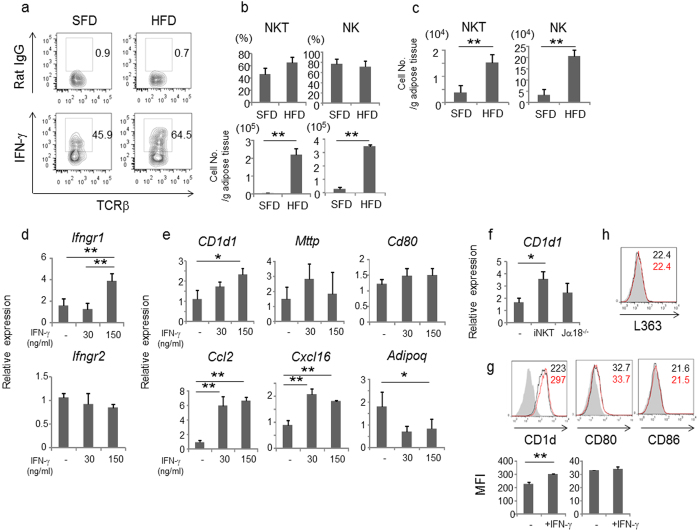
IFN-γ enhances CD1d expression in 3T3-L1 adipocytes. (**a–c**) IFN-γ expression by NKT cells and NK cells in adipose tissue from WT and Jα18^−/−^ mice fed on SFD or HFD (n = 3 in each group). Representative flow cytometric data are shown for NKT cells (gated on TCRβ^+^NK1.1^+^) in WT mice (**a**), the proportion and cell number (/g adipose tissue) of IFN-γ^+^ NKT cells or NK cells (gated on TCRβ^-^NK1.1^+^) in WT mice (**b**), and in Jα18^−/−^ mice (**c**). Intracellular staining was performed after stromal vascular fraction was incubated with PMA/ionomycin in the presence of brefeldin A for 4 h. (**d,e**) The gene expression in 3T3-L1 adipocytes stimulated with IFN-γ (0, 30, 150 ng/ml) for 3 days was quantified by qPCR. (**f**) The expression of *Cd1d1* in 3T3-L1 adipocytes stimulated with the supernatant from 3T3-L1 adipocytes + iNKT cells from WT mice or NKT cells from Jα18^−/−^ mice. (**g,h**) The expression of CD1d, CD80 and CD86 (**g**) and α-GalCer:CD1d complex (**h**), in 3T3-L1 adipocytes stimulated with IFN-γ (gray: isotype, black line: no treatment, red line: IFN−γ treatment) was analyzed by flow cytometry. Numbers are shown as MFI. Representative data from at least 2 independent experiments are shown. Data are shown as mean ± s.d. Statistical analysis was performed according to the Student’s *t*-test and the Tukey-Kramer test. *P < 0.05, **P < 0.01.

To examine the activation of NKT cells in visceral adipose tissue of obese mice, we compared IFN-γ production by NKT cells gated as the TCRβ^+^NK1.1^+^ population that contains both iNKT and vNKT cells in WT mice or by vNKT cells gated similarly in Jα18^−/−^ mice between mice fed a standard fat diet (SFD) and those fed a high-fat diet (HFD). We found that NKT cells in mice fed HFD had an increased IFN-γ^+^ fraction than mice fed SFD (Fig. 5a). The difference appeared significant in TCRβ^+^NK1.1^+^ (iNKT and vNKT) cells but not TCRβ^−^NK1.1^+^ (NK) cells (Fig. 5b, upper panels). When the actual cell numbers per unit adipose tissue weight (g) were compared, significant increases in numbers of IFN-γ-producing TCRβ^+^NK1.1^+^ (iNKT and vNKT) cells and NK cells were detected in mice fed HFD compared with SFD (Fig. 5b, lower panels). Furthermore, high numbers of IFN-γ-producing TCRβ^+^NK1.1^+^ (vNKT) cells and NK cells were detected in Jα18^−/−^ mice fed HFD compared with SFD (Fig. 5c). The above results unequivocally indicated that vNKT cells accumulate in visceral adipose tissue and that these cells produce significant amount of IFN-γ during obesity, although levels produced by iNKT cells compared with vNKT cells appeared higher.

### IFN-γ promotes adipocytes to induce inflammation

To further analyze the effects of IFN-γ produced by iNKT, vNKT and NK cells on adipocytes, we first examined the expression of the IFN-γ receptor and its proximal signaling in adipocytes. 3T3-L1 adipocytes expressed both *Ifngr1* and *Ifngr2* (Fig. 5d). Phosphorylation of STAT1, a proximal component of IFN-γ signaling, was detected at 10 min and was further enhanced at 30 min after IFN-γ stimulation, and phosphorylation at Y701 was significantly blocked by addition of the Jak1/2 inhibitor ruxolitinib (Supplementary Fig. 1a,b). Next, we compared expression of genes relevant to NKT cell function in IFN-γ−treated 3T3-L1 adipocytes. IFN-γ induced *Cd1d1* expression levels, the chemokines *Ccl2* and *Cxcl16*, and microsomal triglyceride transfer protein (*Mttp*) that is important for loading endogenous lipid ligands onto CD1d. Importantly, the expression of *Adipoq* that has anti-inflammatory properties was significantly decreased by IFN-γ treatment (Fig. 5e). Ruxolitinib, an inhibitor of the Jak1/Jak2 kinases, inhibited these effects of IFN-γ treatment on gene expression (Supplementary Fig. 1b,c). CD1d expression was also increased by addition of culture supernatant containing IFN-γ of NKT cells co-cultured with 3T3-L1 adipocytes, suggesting that the interaction between NKT cells and adipocytes further enhances Ag presentation (Fig. 5f). Additionally, IFN-γ increased surface expression of CD1d but not CD80, CD86 (Fig. 5g), or the α-GalCer:CD1d complex (Fig. 5h) in 3T3-L1 adipocytes.

### CD1d^f/f^-adipoq-cre mice are partially protected against diet-induced obesity

**Figure 6:**
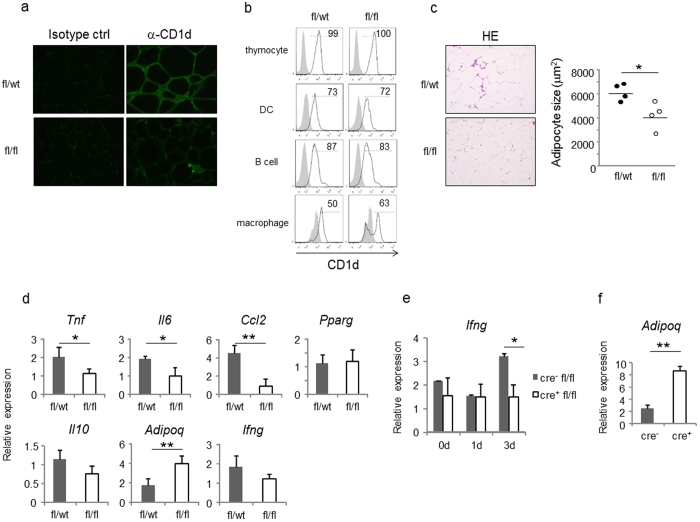
Adipose tissue inflammation is suppressed in CD1d^f/f^-adipoq-cre mice. (**a**) A section of adipose tissue was stained with α-CD1d Ab + Alexa488 α-rat IgG. (**b**) The expression of CD1d in thymocytes (CD4^+^CD8^+^), splenic DC (CD11b^+^CD11c^+^), splenic B cells (B220^+^) and adipose tissue macrophages (CD11b^+^F4/80^+^) (n = 2 in each group). (**c**) A section of adipose tissue was stained with HE and adipocyte size was measured. Each dot shows the average for each mouse. (**d**) The gene expression in adipose tissue after HFD feeding was quantified by qPCR (n = 4 in each group). (**e,f**) The expression of *Ifng* during early phase of HFD feeding (0, 1, 3 days) (**e**) (n = 2–4 in each group) and *Adipoq* at 3 days of HFD feeding (**f**) (n = 3 in each group) was quantified by qPCR. Representative data from at least 3 independent experiments are shown, except for (**f**), which is from one experiment. Data are shown as mean ± s.d. Statistical analysis was performed according to the Student’s *t*-test. *P < 0.05, **P < 0.01.

To examine whether the adipocyte-NKT cell interaction as indicated above played a role in adipose tissue inflammation and development of obesity *in vivo*, CD1d^f/f^-adipoq-cre mice were generated by crossbreeding CD1d1^f/f^ mice^20^ with adiponectin-cre mice^21^. To confirm that CD1d expression was disrupted in adipocytes, visceral fat tissue was stained with anti-CD1d mAb. In CD1d^f/wt^-adipoq-cre mice, adipocytes and cells in crown-like structures (CLS; stronger intensity than adipocytes) were both positive for CD1d (Fig. 6a upper panel), whereas CD1d^f/f^-adipoq-cre mice selectively lacked expression of CD1d in adipocytes (Fig. 6b). Of note, when the visceral fat tissue was stained with hematoxylin and eosin, the size of adipocytes in CD1d^f/f^-adipoq-cre mice appeared smaller (Fig. 6c). Moreover, the expression of inflammatory cytokines and chemokines such as *Tnf*, *Il6* and *Ccl2* in adipose tissue was decreased in CD1d^f/f^-adipoq-cre mice compared with control mice. On the other hand, the expression of *Adipoq* in CD1d^f/f^-adipoq-cre mice was higher than in control mice (Fig. 6d). Expression of *Pparg* and *Il10* was similar between the two groups (Fig. 6d). The expression of *Ifng* tended to be higher in control mice compared with CD1d^f/f^-adipoq-cre mice on HFD for 8 wk. Interestingly, while *Ifng* was detected in control mice at day 3 after HFD feeding, such an increase was absent in CD1d^f/f^-adipoq-cre mice (Fig. 6e). However, *Adipoq* expression was increased in a reciprocal fashion (Fig. 6f).

**Figure 7:**
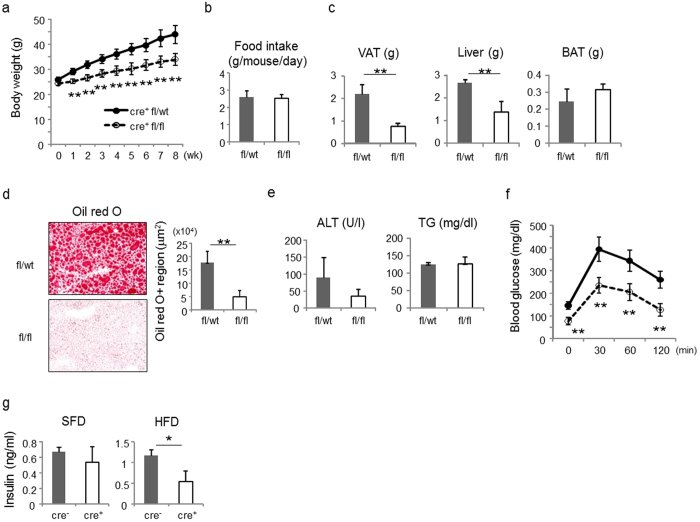
CD1d^f/f^-adipoq-cre mice exhibit suppressed diet-induced obesity and insulin resistance. (**a**) Body weight of CD1d^f/f^-adipoq-cre mice (opened circle) and littermate control mice (closed circle) were fed a high-fat diet (HFD) starting from 8 wk of age and were weighed weekly (n = 4 in each group). (**b**) Food intake (g/mouse/day) in each group. (**c**) The weight of visceral adipose tissue (VAT), liver and brown adipose tissue (BAT) was measured after the 8-wk feeding period. (**d**) A liver section was stained with oil-red-O and the region of lipid droplets (red region) was quantified after the 8-wk feeding period. (**e**) The level of serum ALT (U/l) and TG (mg/dl) was measured after the 8-wk feeding period. (**f**) IPGTT (1 g/kg BW glucose administration) was performed in each group after the 8-wk feeding period. (**g**) The level of serum insulin (ng/ml) in CD1d^f/f^-adipoq-cre mice and CD1d^f/f^ mice fed SFD or HFD for 8 weeks (n = 3 in each group). Representative data from at least 3 independent experiments are shown. Data are shown as mean ± s.d. Statistical analysis was performed according to the Student’s *t*-test. *P < 0.05, **P < 0.01.

CD1d^f/f^-adipoq-cre mice gained less body weight compared with control mice on HFD (Fig. 7a), with no significant difference in the amount of food intake (Fig. 7b). No significant differences were observed when both groups of mice were fed SFD (Supplementary Fig. 2a). The weights of visceral fat and liver but not brown adipose tissue (BAT) in CD1d^f/f^-adipoq-cre mice were also smaller than in control mice (Fig. 7c). Additionally, oil-red-O staining in liver sections showed lower accumulation of lipids, indicating reduced steatosis (Fig. 7d), which was consistent with the levels of ALT, although levels of serum triglycerides were similar (Fig. 7e). Glucose tolerance tests revealed that CD1d^f/f^-adipoq-cre mice maintained insulin sensitivity even when fed HFD (Fig. 7f). Notably, blood glucose levels in CD1d^f/f^-adipoq-cre mice were slightly but significantly lower after 2 hr of IPGTT than in control mice when fed SFD, although fasting blood glucose levels were similar (Supplementary Fig. 2b). The level of serum insulin in CD1d^f/f^-adipoq-cre mice was significantly lower than in control mice fed HFD but not SFD, suggesting that CD1d^f/f^-adipoq-cre mice had improved insulin sensitivity (Fig. 7g).

### Adipocyte-activated NKT cells recruit macrophages to adipose tissue

**Figure 8:**
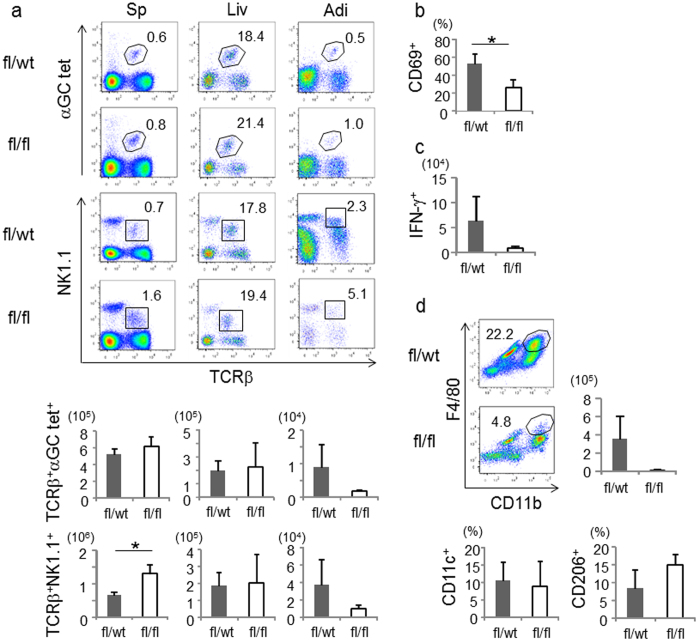
The activation of NKT cells and accumulation of macrophages in CD1d^f/f^-adipoq-cre mice is suppressed. (**a**) Representative flow cytometric data of iNKT cells (TCRβ^+^αGC tet^+^) and NKT cells (TCRβ^+^NK1.1^+^) in spleen, liver and adipose tissue after feeding of HFD for 8 wk. The graph shows the cell number (n = 4 in each group). (**b**) The frequency of CD69^+^ NKT cells in adipose tissue (n = 4 in each group). (**c**) The cell number of IFN-γ^+^ NKT cells in adipose tissue (n = 4 in each group). (**d**) Representative flow cytometric data of macrophages (CD11b^+^F4/80^+^) and cell number. The graph shows the frequency of CD11c^+^ macrophages (M1) and CD206^+^ macrophages (M2) (n = 4 in each group). Representative data from at least 2 independent experiments are shown. Data are shown as mean ± s.d. Statistical analysis was performed according to the Student’s *t*-test. *P < 0.05, **P < 0.01.

To further analyze the mechanism of adiposity suppression in CD1d^f/f^-adipoq-cre mice, we analyzed NKT cells. As CD1d expression in thymocytes and other immunocompetent cells was similar between CD1d^f/f^-adipoq-cre and control mice (Fig. 6b), NKT cells developed normally in CD1d^f/f^-adipoq-cre mice in thymus, spleen, liver and adipose tissue (Fig. 8a and Supplementary Fig. 2c). Cytokine secretion upon α-GalCer administration was also similar to control mice, indicating that there was no defect in iNKT cell function (Supplementary Fig. 2d). Of note, the frequency of iNKT cells (TCRβ^+^αGC/tet^+^) and total NKT cells (TCRβ^+^NK1.1^+^) in spleen, liver and adipose tissue appeared higher in CD1d^f/f^-adipoq-cre mice compared with control mice on HFD (Fig. 8a, upper panel). Likewise, cell numbers of the NKT cell population in spleen and liver were increased in CD1d^f/f^-adipoq-cre, although cell numbers were decreased in adipose tissue, probably because CD1d expression on adipocytes influences cellular infiltration (Fig. 8a, lower panel). The activation marker CD69 was expressed at lower levels on NKT cells in adipose tissue from CD1d^f/f^-adipoq-cre mice compared with control mice (Fig. 8b), and similar findings were observed in spleen and liver. IFN-γ-producing TCRβ^+^NK1.1^+^ cells that include both iNKT and vNKT cells in adipose tissue were also decreased in CD1d^f/f^-adipoq-cre mice compared with control mice, implying that NKT cells were activated by adipocytes and produced IFN-γ when adipocytes expressed CD1d (Fig. 8c). Additionally, fewer macrophages were recruited into adipose tissue in CD1d^f/f^-adipoq-cre than control mice (Fig. 8d). We were unable to detect significant differences in the expression of M1/M2 surface markers (CD11c^+^/CD206^+^) (Fig. 8d) and *Nos2* and *Arg1* genes (data not shown). These results suggested that NKT-adipocyte interactions induce NKT cell activation and recruitment of macrophages that leads to adipose tissue inflammation and promotes DIO.

## Discussion

In the first part of the present study, we have demonstrated that 3T3-L1 adipocytes can present both exogenous and endogenous lipid antigens on CD1d to activate NKT cells, which is consistent with previous reports^23^,^24^. As 3T3-L1 cells express CD1d upon differentiation, mature adipocytes *in vivo* express high levels of CD1d. Moreover, CD80 but not CD86 is significantly induced when mice are fed HFD. Although both iNKT cells from WT or vNKT cells from Jα18^−/−^ mice responded to 3T3-L1 adipocytes in the absence of an exogenous NKT cell ligand, they stained negative with L363 mAb that is thought to recognize α-GlyCer/CD1d complexes^25^. This negative result does not necessary mean that adipocytes are unable to produce and present α-GlyCer on CD1d, but that levels are too low for detection by flow cytometry. Alternatively, it is possible that adipocytes present unique Ag that is not recognized by L363. The Ag, however, is likely to be lipid molecule endogenously synthesized or distributed in adipocytes, since Rakhshandehroo *et al*. demonstrated that the activation of iNKT cells by 3T3-L1 adipocytes required the expression and intact function of microsomal triglyceride transfer protein (MTP)-B that is significantly expressed in mature adipocyte^24^.

In the second part of the present study, we provided evidence that NKT-adipocyte interactions are vital for promoting obesity. We found that adipocyte-specific CD1d deletion abrogated NKT cell activation via adipocytes and thus ameliorated inflammation in adipose tissue, resulting in reduced obesity and improved insulin sensitivity. Our previous study demonstrated that CD1d^−/−^ mice gained less visceral adipose tissue and body weight than WT mice, and exhibited improved insulin sensitivity when fed a HFD^18^. We have shown in the present study that selective disruption of CD1d in adipocytes could reproduce these findings, suggesting that adipocytes are the most critical cell type expressing CD1d for the observed effects on obesity and inflammation.

NKT cells from Jα18^−/−^ mice including vNKT cells in adipose tissue preferentially produced IFN-γ when co-cultured with 3T3-L1 adipocytes (Fig. 3b). In addition, these cells were skewed towards production of Th1 type cytokines compared with those in spleen and liver (data not shown). Other reports have suggested that iNKT cells in adipose tissue have a regulatory role by producing IL-10 (NKT10 cells)^26^,^27^, suggesting that adipose tissue NKT cells constitute a specialized subset of NKT (atNKT) cells. In the studies where iNKT cells exhibited beneficial roles in obesity-associated inflammation, Jα18^−/−^ mice demonstrated more gain of body weight when fed HFD^15^,^16^,^23^. The reasons for these divergent findings remain unclear.

We observed that NKT and NK cells produced IFN-γ and accumulated in adipose tissue in mice on HFD. Our *in vitro* studies showed that IFN-γ enhanced CD1d and CCL2 expression levels but decreased adiponectin expression in 3T3-L1 adipocytes in a STAT1-dependent manner, indicating that IFN-γ plays a key pro-inflammatory role in adipose tissue. Other reports have shown that IFN-γ attenuates insulin signaling due to down-regulation of the insulin receptor, insulin receptor substrate-1 and GLUT4 in human SGBS adipocytes via the JAK-STAT1 pathway^28^. IFN-γ-deficient mice exhibited reduced gain of body weight, improved glucose tolerance and hepatic insulin sensitivity even when fed a low-fat diet^29^. Furthermore, IFN-γ-deficiency limited inflammatory cell accumulation in adipose tissue associated with a reduction in TNF-α, CCL2 and RANTES expression^30^. Thus, IFN-γ produced by NKT cells is a critical regulator for the development of obesity, and its production by iNKT cells, vNKT cells and downstream effector or amplifier cells, NK cells, is controlled through antigen presentation by adipocytes that express CD1d. In contrast to the present findings, Wensveen *et al*. demonstrated that IFN-γ produced by NK cells through activation of natural cytotoxicity triggering receptor (NCR)1 with a putative ligand expressed on adipocytes, induced M1 macrophage polarization and enhanced adipose tissue inflammation and insulin resistance^31^. Lee *et al*. demonstrated that adipose NK cells appeared to be upstream regulators of macrophage and adipocyte functions^32^. These reports imply that adipose NK cells are one of the key players that modulate the immune balance in adipose tissue.

Macrophages are closely associated with chronic inflammation in adipose tissue, and the CCL2/CCR-2 pathway is crucial in signaling macrophage recruitment into adipose tissue during obesity^33^. We found that macrophage numbers were decreased 78.3% in CD1d^f/f^-adipoq-cre mice compared with control mice, likely due to reduction of CCL2 in these animals, suggesting that adipocyte-NKT cell interactions are critical for recruitment of macrophages in adipose tissue during obesity (Fig. 8d). M2 macrophages (anti-inflammatory) expressing IL-10, CD206 and arginase1 are present in normal tissues at high levels whereas M1 macrophages (pro-inflammatory) expressing TNF-α, IL-6, CCL2 and iNOS are prevalent in inflamed tissues^34^. In the present study we did not detect clear differences in the polarization of macrophages in adipose tissue between CD1d^f/f^-adipoq-cre and control mice on HFD. It is possible that the 8 wk of HFD feeding we employed was too short to substantially alter macrophage differentiation, because previous studies detected significant differences only after 12 wk of HFD feeding^4^,^31^. Notably, selective CD1d depletion on adipocytes appeared to suppress the development of obesity prior to macrophage polarization.

While it has been shown that CD1d disruption both globally^18^,^19^ and selectively in adipocytes ameliorates the development of obesity, other research groups have reported that CD1d disruption enhances the development of obesity and that iNKT cells in adipose tissue produce IL-4 and IL-10 that induce M2 macrophages^15^,^16^,^17^. There may be several reasons for these divergent outcomes in the development of obesity, including differences in the mouse strains used, diets (origins of fat) fed, microbiomes in the guts (*i.e.*, environments of animal facilities), and combinations of these variables. For example, we have used an HFD containing beef tallow and safflower oil as the fat source whereas other labs used lard or soybean oil as the fat source. The endogenous ligands for NKT cells in adipocytes might be influenced by the chemical properties of the fat source employed and thus influence NKT cell function. Further investigations such as analyses of intestinal flora will be needed to elucidate the reasons for the divergent results obtained.

Our present findings demonstrate that direct interactions between NKT cells and adipocytes contribute to immune regulation in adipose tissue. Regulation of the expression of endogenous ligands of NKT cells in adipocytes and modulation of NKT cell activation in adipose tissue have important implications for developing novel therapeutic approaches for various disorders of lipid metabolism such as obesity and the metabolic syndrome.

## Methods

### Mice

C57BL/6 mice were purchased from CLEA Japan, Inc. Adiponectin-cre mice (B6;FVB-Tg (Adipoq-cre)1Evdr/J; TJL stock number: 010803)^20^ and CD1d1-floxed mice (C57BL/6-Cd1d1^tm1.1Aben^/J; TJL stock number: 016929)^21^ were purchased from The Jackson Laboratory. CD1d1^−/−^ and Jα18^−/−^ were used as NKT cell-deficient mice^35^,^36^. All mice were maintained under specific pathogen-free conditions on food and water *ad libitum* in the animal facility at Kitasato University School of Medicine. In diet-induced obesity experiments, mice were fed either regular chow as a standard fat diet (SFD) or a high fat diet (HFD) (CLEA Japan HFD-32: 32% fat (powdered tallow and safflower oil of high oleic type), 25.5% protein, 2.9% fiber, 4.0% minerals, 29.4% nitrogen, and 6.2% water) starting from 8 wk of age. All experimental procedures on mice were conducted in accordance with the protocol approved by the Animal Experimentation and Ethics Committee of Kitasato University School of Medicine (#2014–141, #2015–063).

### Cell culture

3T3-L1 fibroblasts, obtained from the American Type Culture Collection (ATCC CCL-92.1), were cultured in high glucose DMEM (Sigma) containing 10% FCS, 50 μM β-mercaptoethanol, 100 units/ml penicillin, and 100 μg/ml streptomycin (pre-adipocyte medium). Two-day post-confluent cells were incubated in differentiation medium (pre-adipocyte medium containing 5 μg/mL insulin (Eli Lilly), 0.5 mM 3-isobutyl-1-methylxanthine (IBMX; Sigma), and 0.25 μM dexamethasone (Sigma)) for 2 days. Then the cells were placed in insulin-containing medium (pre-adipocyte medium containing 5 μg/mL insulin) for 2 days. The media was replaced every 2 days with insulin-containing medium. The cells were ready to be used in experiments on day 7 to 10 after differentiation. For examination of the accumulation of lipid droplets, 3T3-L1 adipocytes were stained with oil-red-O (Sigma). In co-culture experiments, 3T3-L1 cells were cultured on plates coated with collagen gel (Nitta Gelatin Inc.). Differentiated 3T3-L1 adipocytes were incubated with 5 × 10^5^ 2E10 NKT cell hybridomas or 2 × 10^4^ sorted NKT cells in the presence of IL-2 (40 ng/ml, BioLegend) and in some experiments α-GalCer (100 ng/ml, Kyowa Hakko Kirin Co., Ltd.). Anti-CD1d mAb (clone 1B1; 20 μg/ml or 50 μg/ml, BioLegend) was used for blocking experiments, and recombinant IFN-γ (15–150 ng/ml, PEPROTECH), TNF-α (2 ng/ml, PEPROTECH) and ruxolitinib (0.1–3 μM, INCB018424, Selleckchem) were used in some cultures.

### Cell surface staining

Single cell suspensions from spleen, liver and visceral adipose tissue were incubated with 2.4G2 mAb (anti-FcγRIII/II) to block non-specific binding of primary mAb and then cells were stained with a combination of the following mAb: FITC conjugated anti-TCRβ (H57-597, BioLegend), anti-CD11b (Mac-1, TONBO Biosciences), anti-CD206 (C068C2, BioLegend), APC conjugated anti-NK1.1 (PK136, BioLegend), anti-CD11c (HL3, BD Pharmingen), PE conjugated anti-α-GalCer:CD1d complex antibody (L363, BioLegend), α-GalCer (PBS-57)-loaded CD1d tetramer kindly provided by NIH Tetramer Core Facility at Emory University (Atlanta) and Brilliant Violet 421 conjugated anti-F4/80 (BM8.1, TONBO Biosciences). 3T3-L1 adipocytes were stripped off by PBS containing 1.5 mM EDTA and stained with PE conjugated anti-CD1d (1B1, BioLegend), anti-CD80 (16-10A1, BD Pharmingen) and -CD86 (GL1, BD Pharmingen). Stained cells were sorted using FACS Aria (BD Biosciences) or assessed using FACS Caliber flow cytometers (BD Biosciences). Data were analyzed with FlowJo software (FlowJo, LLC).

### Intracellular cytokine staining

Mononuclear cells from stromal vascular fraction (SVF) were isolated from the digest of visceral adipose tissue by mincing by scissors and incubating with collagenase D solution (2 mg/ml, Roche) for 1–1.5 h at 37 °C. Collected cells from the pellet fraction after centrifugation at 1200× *g* for 5 min were incubated with PMA (10 ng/ml, Sigma), ionomycin (1 μg/ml, Sigma) and brefeldin A (5 μg/ml, BioLegend) for 4 h. Then cells were washed and stained for surface marker expression before fixation and permeabilization using the FOXP3 Fix/Perm Buffer Set (BioLegend) according to the manufacturer’s protocol. Thereafter, treated cells were stained with PE conjugated anti-IFN-γ mAb (XMG1.2, BioLegend). Stained cells were acquired with FACS Verse flow cytometers (BD Biosciences) and analyzed with FlowJo software (FlowJo, LLC).

### Quantification of cytokines

The concentration of Th1/Th2/Th17 and inflammatory cytokines, including IFN-γ, tumor necrosis factor (TNF)-α, IL-1α, 2, 4, 5, 13, 6, 10, 17, 21, 22, 27, and GM-CSF in culture supernatant was quantified with Mouse Th1/Th2/Th17/Th22 13 plex Kit FlowCytomix or 10 plex kit FlowCytomix (Affymetrix) according to the manufacturer’s protocol with a flow cytometer.

### Quantitative real-time PCR

Total RNA was extracted using Trizol reagent (Life Technologies). cDNA was synthesized using PrimeScript RT Master Mix (TaKaRa) with total RNA. Real-time PCR was performed using SYBR Premix Ex Taq II (TaKaRa) and CFX96 real time PCR detection system (Bio-Rad) according to the manufacturer’s protocol. Target gene expression was normalized with β-actin and calculated using the 2−ΔΔCT method. Primers were as follows: *Actb* (forward 5′-GGCTGTATTCCCCTCCATCG-3′; reverse 5′-CCAGTTGGTAACAATGCCATGT-3′), *Cd1d1* (forward 5′-ACTCAGCCACCATCAGCTTC-3′; reverse 5′-AGGGTACATTTCACAGCCCG-3′), *Cd80* (forward 5′-CCTGGCTTTCCCCATCATGT-3′; reverse 5′-AGAGTTGTAACGGCAAGGCA-3′), *Cd86* (forward 5′-CTTACGGAAGCACCCACGAT-3′; reverse 5′-CAACTTTTGCTGGTCCTGCC-3′), *Pparg* (forward 5′-ATGGAGCCTAAGTTTGAGTTGC-3′; reverse 5′-TGTCCTCGATGGGCTTCA-3′), *Fabp4* (forward 5′-TGAAATCACCGCAGACGACA-3′; reverse 5′-GGCCTCTTCCTTTGGCTCAT-3′), *Mttp* (forward 5′-CTTCTTCATCTGGTCCGGGG-3′; reverse 5′-CCAGACCGCTCAATTTTGCA-3′), *Ccl2* (forward 5′-CCCAATGAGTAGGCTGGAGA-3′; reverse 5′-GCTAAGACCTTAGGGCAGA-3′), *Cxcl16* (forward 5′-CCTTGTCTCTTGCGTTCTTCC-3′; reverse 5′-TCCAAAGTACCCTGCGGTATC-3′), *Adipoq* (forward 5′-ACAGGAGATGTTGGAATGACAG-3′; reverse 5′-CTGCCGTCATAATGATTCTGTT-3′), *Ifngr1* (forward 5′-GACGAGCACTGAGGATCCTG-3′; reverse 5′-CTTTAACTCTGGCCCAGGCA-3′), *Ifngr2* (forward 5′-CCTGATTCCGTTGGGCATCT-3′; reverse 5′-CCGTCCTTGTCCAAGACCTC-3′), *Il6* (forward 5′-CACATGTTCTCTGGGAAATCG-3′; reverse 5′-TTGTATCTCTGGAAGTTTCAGATTGTT -3′), *Tnf* (forward 5′-GCCACCACGCTCTTCTGTCTAC-3′; reverse 5′-GGGTCTGGGCCATAGAACTGAT-3′), *Il10* (forward 5′-GCTCTTACTGACTGGCATGAG-3′; reverse 5′-GCGAGCTCTAGGAGCATGTG-3′) and *Ifng* (forward 5′-TCAAGTGGCATAGATGTGGAAGAA-3′; reverse 5′-TGGCTCTGCAGGATTTTCATG-3′) (Hokkaido System Science).

### Western Blotting

3T3-L1 adipocytes were treated with IFN-γ for the time periods indicated in figure legends and ruxolitinib was added for inhibition experiments 1 hr prior to IFN-γ treatment. Whole cell lysates were prepared using NP-40 surfactant buffer and 10–20 μg protein of total cell lysates was separated by SDS-PAGE (10% Mini-PROTEAN Precast Gels, Bio-Rad), then blotted onto PVDF membranes. Antibodies specific for STAT1 (1:1000) and phosphorylated STAT1 (1:1000) (PhosphoPlus^®^ Stat1 (Tyr701) Antibody Kit, Cell Signaling TECHNOLOGY) were used according to the manufacturer’s protocol. As a control, anti-β-actin Ab (1:1000) (BioLegend) was used. Proteins were detected with peroxidase-coupled secondary antibodies (1:1000) using an ECL substrate, and visualized by ImageQuant LAS 4000 system (GE Healthcare).

### Histology and quantitative analyses of microscopic images

Visceral adipose tissue was fixed with buffered formaldehyde solution (10%) followed by standard protocol for paraffin-embedded sections and hematoxylin-eosin (HE) staining. Images of the HE-stained adipose tissue were incorporated with a BIOREVO microscope (BZ-9000; KEYENCE), and morphometric analyses were performed with image analysis software (BZ-II) equipped on the microscope. Liver samples were snap-frozen in OCT compound (Sakura Finetek) with liquid nitrogen, and the cryosections were stained with Oil-Red-O (ORO) (Sigma). Images of lipid droplets in hepatocytes stained red were also quantified by image analysis software (BZ-II).

### Blood chemistry

Triglyceride (TG) and alanine aminotransferase (ALT) concentrations in sera were quantified by colorimetric assays using DRI-CHEM 7000 V (FUJIFILM) according to the manufacturer’s protocol. The level of insulin in sera was quantified by mouse insulin ELISA kit (Morinaga) according to the manufacturer’s protocol.

### Glucose tolerance test

Intraperitoneal (i.p.) glucose tolerance test (IPGTT) was performed by i.p. injection of glucose solution (1 g/kg) after 16 h of fasting. The blood glucose level was serially quantified with a blood glucose monitor using MEDISAFE MINI (TERUMO Corp.).

### Statistics

Results are presented as means ± standard deviation (s.d.). Statistical analysis between two groups was performed by Student’s *t*-test and among three groups was performed using ANOVA followed by Tukey-Kramer tests. Values with *P* < 0.05 were considered statistically significant.

## Additional Information

**How to cite this article**: Satoh, M. *et al*. Adipocyte-specific CD1d-deficiency mitigates diet-induced obesity and insulin resistance in mice. *Sci. Rep.* **6**, 28473; doi: 10.1038/srep28473 (2016).

## Supplementary Material

Supplementary Information
