# Supplementary material for: Adipocyte-specific CD1d-deficiency mitigates diet-induced obesity and insulin resistance in mice
Source: Sci Rep. 2016 Jun 22;6:28473. doi: 10.1038/srep28473 (PMC4916414; doi:10.1038/srep28473)
Supplement: Supplementary Information [file srep28473-s1.pdf]

Adipocyte-specific CD1d-deficiency mitigates diet-induced obesity and insulin resistance in mice

Masashi Satoh[1], Miyuki Hoshino[1], Koki Fujita[1], Misao Iizuka[1], Satoshi Fujii[2], Christopher S. Clingan[3], Luc Van Kaer[4], Kazuya Iwabuchi[1*]

Supplemental Figure 1. The IFN-γ-STAT1 axis modulates gene expression in 3T3-L1 adipocytes

(a) Phosphorylated stat1 (p-stat1), total stat1 and β-actin (control) in 3T3-L1 adipocytes stimulated by IFN-γ treatment (15 ng/ml) was detected by Western blot. (b) Inhibition of stat1 phosphorylation by ruxolitinib. (c) Modulation of gene expression in 3T3-L1 adipocytes by IFN-γ (30 ng/ml) and ruxolitinib (3 μM) treatment for 3 days. Ruxolitinib treatement was initiated 1 h before IFN-γ treatment. Representative data from at least 2 independent experiments are shown. Data are shown as mean ± s.d. Statistical analysis was performed according to the Tukey-Kramer test. *$P < 0.05$, **$P < 0.01$.

Supplemental Figure 2. Analysis of CD1d$^{f/f}$-adipoq-cre mice on SFD

(a) Body weights of CD1d$^{f/f}$-adipoq-cre mice (closed circle) and littermate control mice (opened circle) fed a SFD for 8 wk and weighed weekly (n=4-5 in each group). (b) IPGTT (1 g/kg BW glucose administration) was performed in each group after SFD feeding for 8 wk (n=4-5 in each group). (c) Representative flow cytometric data of iNKT cells (TCRβ^{+}αGC tet^{+}) and NKT cells (TCRβ^{+}NK1.1^{+}) from thymus, spleen, liver and adipose tissue in mice fed SFD at 8 wk of age (n=3-4 in each group). (d) Serum cytokines in CD1d$^{f/f}$-adipoq-cre mice or littermate control mice following α-GalCer treatment (2 μg/mouse, i.v.) (n=3 in each group). Representative data from at least 2 independent experiments are shown. Data are shown as mean ± s.d. Statistical analysis was

performed according to the Student's *t*-test. *$P < 0.05$, **$P < 0.01$.

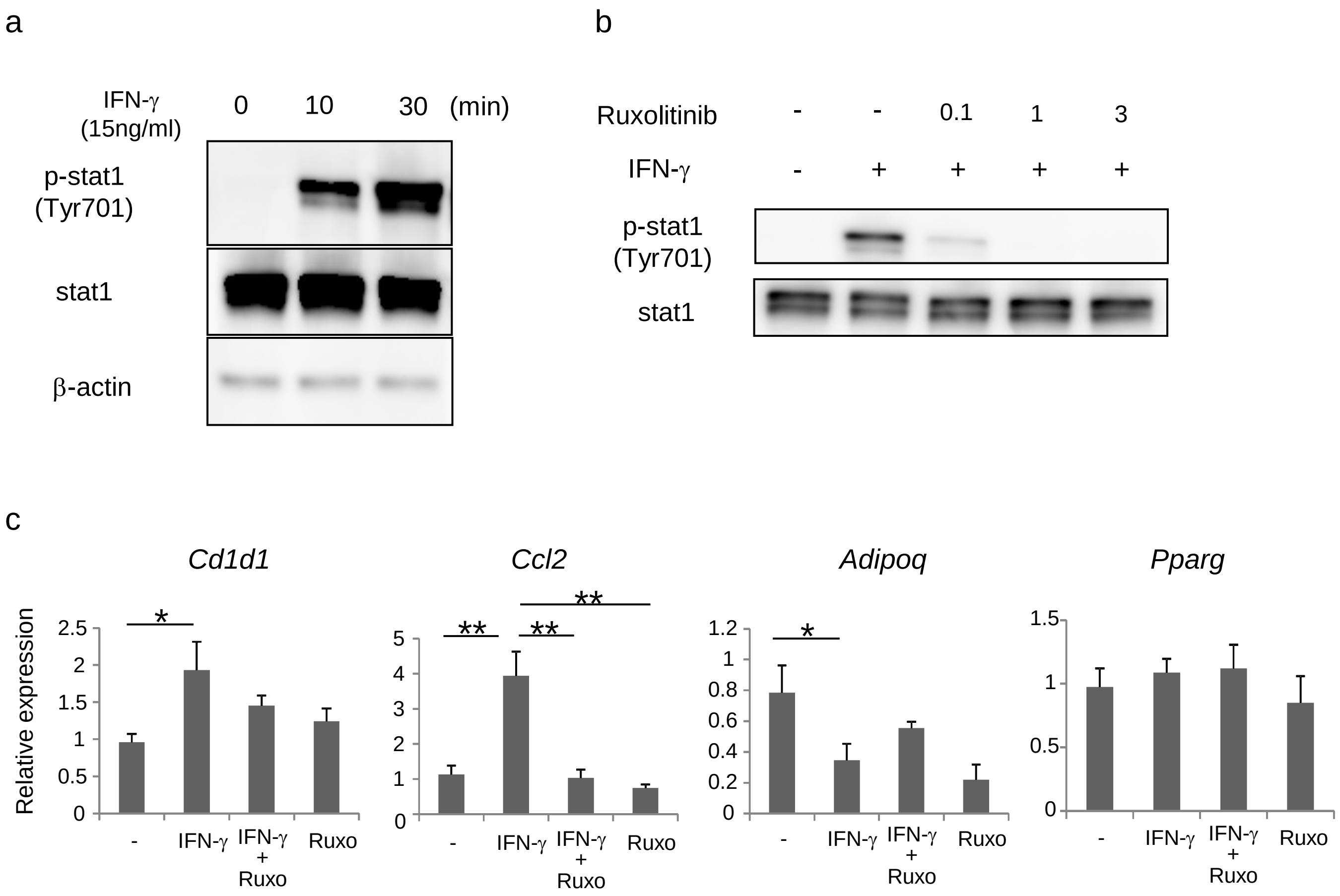
a
IFN-γ (15ng/ml)
0
10
30
(min)
p-stat1 (Tyr701)
stat1
β-actin
b
Ruxolitinib
-
-
0.1
1
3
IFN-γ
-
+
+
+
+
p-stat1 (Tyr701)
stat1
c
Cd1d1
Ccl2
Adipoq
Pparg
Relative expression
2.5
2
1.5
1
0.5
0
5
4
3
2
1
0
1.2
1
0.8
0.6
0.4
0.2
0
1.5
1
0.5
0
*
**
**
**
*
-
IFN-γ
IFN-γ + Ruxo
Ruxo

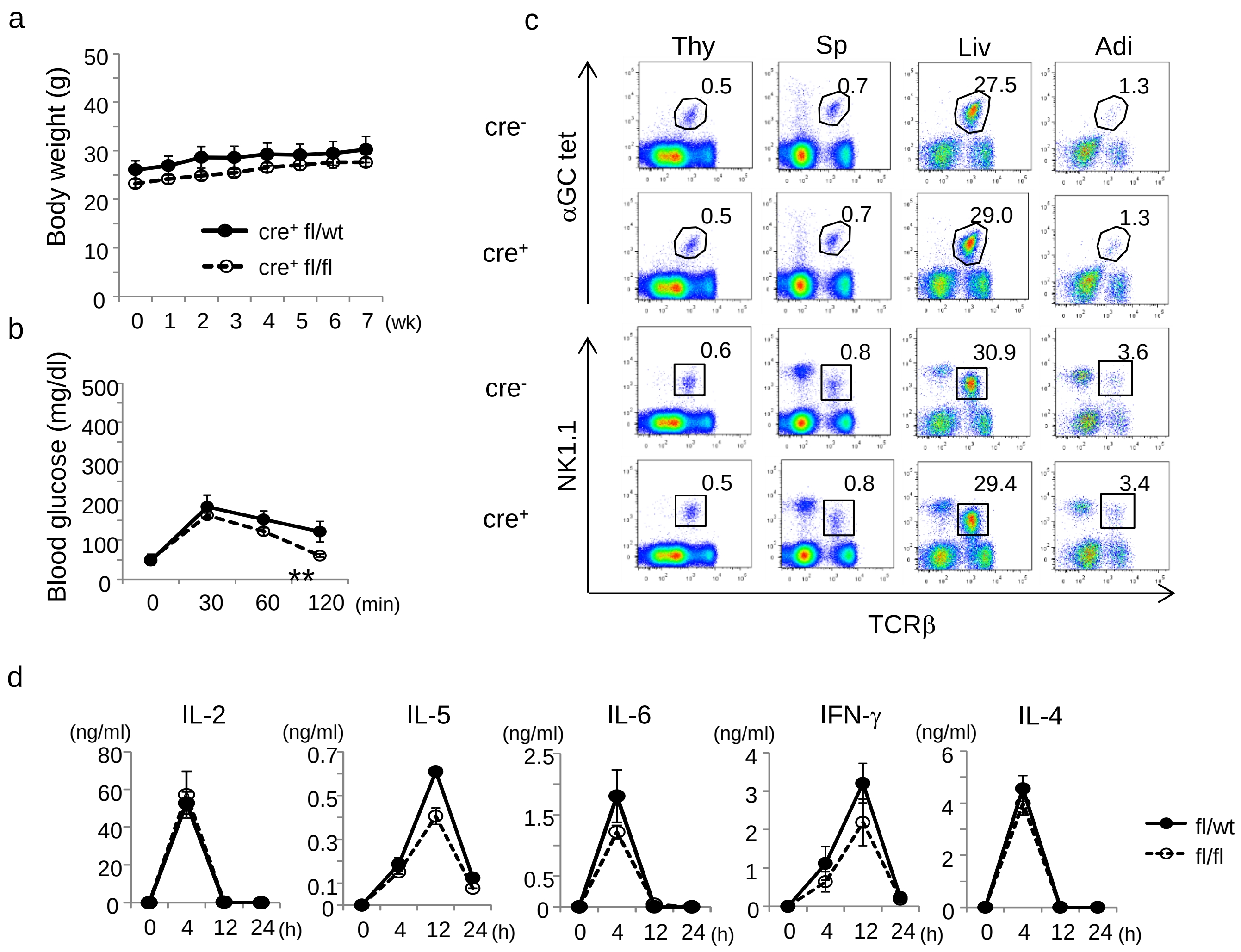

a
Body weight (g)
50
40
30
20
10
0
0 1 2 3 4 5 6 7 (wk)
cre+ fl/wt
cre+ fl/fl
b
Blood glucose (mg/dl)
500
400
300
200
100
0
0 30 60 120 (min)
**
c
Thy
Sp
Liv
Adi
cre-
cre+
αGC tet
NK1.1
TCRβ
0.5
0.7
27.5
1.3
0.5
0.7
29.0
1.3
0.6
0.8
30.9
3.6
0.5
0.8
29.4
3.4
d
IL-2
IL-5
IL-6
IFN-γ
IL-4
(ng/ml)
0 4 12 24 (h)
fl/wt
fl/fl
